# Genetic and Quantitative Trait Locus Analysis of Cell Wall Components and Forage Digestibility in the Zheng58 × HD568 Maize RIL Population at Anthesis Stage

**DOI:** 10.3389/fpls.2017.01472

**Published:** 2017-08-24

**Authors:** Kun Li, Hongwu Wang, Xiaojiao Hu, Feiqian Ma, Yujin Wu, Qi Wang, Zhifang Liu, Changling Huang

**Affiliations:** Institute of Crop Sciences, Chinese Academy of Agricultural Sciences Beijing, China

**Keywords:** QTL, maize, cell wall, lignin, digestibility

## Abstract

The plant cell wall plays vital roles in various aspects of the plant life cycle. It provides a basic structure for cells and gives mechanical rigidity to the whole plant. Some complex cell wall components are involved in signal transduction during pathogenic infection and pest infestations. Moreover, the lignification level of cell walls strongly influences the digestibility of forage plants. To determine the genetic bases of cell wall components and digestibility, quantitative trait locus (QTL) analyses for six related traits were performed using a recombinant inbred line (RIL) population from a cross between Zheng58 and HD568. Eight QTL for *in vitro* neutral detergent fiber (NDF) digestibility were observed, out of which only two increasing alleles came from HD568. Three QTL out of ten with alleles increasing *in vitro* dry matter digestibility also originated from HD568. Five–ten QTL were detected for lignin, cellulose content, acid detergent fiber, and NDF content. Among these results, 29.8% (14/47) of QTL explained >10% of the phenotypic variation in the RIL population, whereas 70.2% (33/47) explained ≤10%. These results revealed that in maize stalks, a few large-effect QTL and a number of minor-effect QTL contributed to most of the genetic components involved in cell wall biosynthesis and digestibility.

## Introduction

The plant cell wall is an amorphous matrix that surrounds the cell membrane. Plant cell walls provide the basic mechanical support that allows plants to stand upright. Moreover, microbial infection usually induces lignification of the cell wall, which protects plants from further harm. Cell wall architecture plays important roles in plant responses to various abiotic stresses, such as drought, flooding, heat, cold, and salt and is essential in stress sensing and signal transduction (Gall et al., 2015). Furthermore, the cell walls of forage plants are the main resources for animal feeding. Lignocellulose biomass is also considered to be a source of renewable energy for the production of biofuel (Bhalla et al., 2013).

From a livestock feeding perspective, the primary goal is to focus on good forage quality, which is usually defined as a high forage intake and digestibility. The plant cell wall is a composite material that consists mainly of cellulose (CEL), hemicellulose, and lignin (LIG) (Chen, 2014). These three organic compounds are also major components of plant fiber and forage dry matter. Since the concentrations of cell wall constituents are correlated with forage intake and digestibility, they are regarded as the most important factors in forage utilization (Paterson et al., 1994). Previous research has demonstrated that each cell wall component has a specific digestibility. The digestibility of CEL ranges from 50 to 90%, with hemicellulose being 20–80% digestible (Kebede et al., 2016; and reference therein). Thus, to evaluate the forage quality of plants, several chemical analysis methods have been introduced to measure the cell wall contents. Fiber content is usually quantified as neutral detergent fiber (NDF) and acid detergent fiber (ADF), whereas LIG content can be measured as acid detergent lignin (ADL). Neutral detergent fiber mainly consists of CEL, hemicellulose, and LIG (Krakowsky et al., 2003). After hemicelluloses are solubilized by treatment with an acid detergent, CEL and LIG, which mainly comprise ADF, are left in the residue of the cell wall (Truntzler et al., 2010). Consequently, hemicellulose content can be determined by NDF minus ADF, and CEL content is assumed to be the difference between ADF and ADL (Jess, 1986; Truntzler et al., 2010). Although LIGs are the most difficult components for microorganisms in the rumen of an herbivore to digest, the correlations between digestibility and lignification have been shown to vary according to the genetic background of the plant (Barrière et al., 2003). Moreover, the LIG levels in maize and other species were not shown to be well-correlated with the enzyme-mediated digestibility of the cell wall (Marriott et al., 2014; Penning et al., 2014; Guzzo de Carli Poelking et al., 2015; Cass et al., 2016). In addition to the contents of specific cell wall components, the associations and cross-linkages between polysaccharides and LIGs also contribute to the digestibility of cell wall (Truntzler et al., 2010). It seems impossible to improve the forage quality by selecting only for low amount of LIGs or other cell wall components. Measuring forage and cell wall digestibility directly allows breeders to evaluate the digestibility level of the genetic germplasm. Since *in vivo* methods for detecting digestibility are complex and expensive for breeding programs, *in vitro* methods for estimating digestibility, which include *in vitro* neutral detergent fiber digestibility (IVNDFD) and *in vitro* dry matter digestibility (IVDMD) methods (Marten and Barnes, 1979; Hatfield et al., 1994), have been introduced into forage analyses. In addition, the introduction of near-infrared reflectance spectroscopy (NIRS) provides rapid estimates of cell wall components and digestibility at lower costs and with greater accuracy (Shenk and Westerhaus, 1994).

Through forward genetic screening, a set of brown-midrib maize mutants that showed decreased LIG contents and improved digestibility by ruminant animals were discovered (Cherney et al., 1991; Barriere and Argillier, 1993; Marita et al., 2003; Vermerris et al., 2007). However, the modification of one monolignol-related gene in these mutants causes larger changes than expected in the cell wall polymers (Courtial et al., 2013). Moreover, the application of the brown-midrib mutants in improving forage quality also shows a negative effect on biomass yield-related traits (Li and Chapple, 2010; Simmons et al., 2010). Therefore, LIG pathway mutants cannot be successfully used to improve forage digestibility due to their side effects. Breeding for high digestibility in forage maize with marker-assisted selection (MAS) is an alternative approach for improving forage quality. Dissecting the genetic basis of cell wall-related traits has greatly influenced the understanding of the biosynthetic pathways of cell wall components and has provided useful molecular markers for MAS in forage breeding. Over the last two decades, quantitative trait locus (QTL) analyses of the composition and digestibility traits of the cell wall have been performed in maize (Lübberstedt et al., 1997a,b; Bohn et al., 2000; Barriere et al., 2001; Méchin et al., 2001; Papst et al., 2001; Roussel et al., 2002; Cardinal et al., 2003; Fontaine et al., 2003; Krakowsky et al., 2003, 2005, 2006; Barrière et al., 2007; Riboulet et al., 2008); these studies have identified more than 400 QTL across the maize genome. However, the use of a small number of early generation markers, such as Restriction Fragment Length Polymorphism (RFLP) and Simple Sequence Repeats (SSR), caused low resolution in some previous studies. With the development of genotyping technologies, single nucleotide polymorphism (SNP) markers have been widely used in linkage and association studies in maize (Li et al., 2012, 2013, 2014; Shutu et al., 2012; Peiffer et al., 2014). Compared with SSR markers, SNPs are more accurate, less time-consuming, and less costly to identify; moreover, SNPs are more useful for improving the resolution of genetic mapping (Yan et al., 2010; Yang et al., 2011; Shutu et al., 2012). Currently, SNP genotyping is usually performed using DNA chips (Yan et al., 2009, 2010; Ganal et al., 2011; Li et al., 2014) and genotype-by-sequencing (GBS) approaches (Gore et al., 2009; Lai et al., 2010; Chia et al., 2012; Jiao et al., 2012; Bukowski et al., 2015).

In most of the previous QTL mapping studies of cell wall component and digestibility traits were performed at the silage stage. Whereas cell wall components accumulation in plant is a dynamic process. In this process, a lot of genes or locus function in cell wall component biosynthesis in different organs and growth stages. Besides silage stage, anthesis stage is also a key period for forage maize growth, which leads a change from vegetative growth to reproductive growth. In this study, a maize Zheng58 × HD568 recombinant inbred line (RIL) population was developed and genotyped using a GoldenGate maize SNP assay, which contains 3,072 SNPs. Our objectives were to identify QTL associated with the cell wall composition and digestibility traits of maize stalks at the anthesis stage and dissect the genetic architecture of the target traits evaluated herein.

## Materials and Methods

### Germplasm and Field Experiments

A RIL population consisting of 220 lines was developed by single seed descent (SSD) up until the F10 generation in a cross between inbred lines Zheng58 and HD568, which are the parental lines of the elite commercial hybrid Zhongdan909 in China. Zheng58 originated from BSSS group, HD568 came from Tangsipingtou (TSPT) germplasm which is a traditional heterotic group used in maize breeding of China. All F10 RILs and the two parents were planted in a randomized complete block design with two replicates in Hainan in 2012 and Beijing in 2013. Each line was grown in a single 2.5 m row with 0.67 m between rows and a planting density of 45,000 plants/ha.

### Phenotyping Methods

At the anthesis stage, the second–fifth internodes above the ground were collected from six plants of each line. All samples were immediately enzyme deactivated at 105°C for 30 min in a forced air oven and air-dried for 10–14 days. Dried stalk samples were ground with a mill and filtered through a screen with a mesh size of 0.1 mm. Cellulose, LIG, ADF, NDF, and IVDMD were estimated using NIRS. Before the measurements, the stalk samples were dried at 45°C for 48 h to exclude the influence of moisture. The samples were scanned through a near-infrared reflectance spectrophotometer (VECTOR22/N; BURKER Optik, Ettlingen, Germany). Cellulose, LIG, ADF, NDF, and IVDMD were determined using NIRS prediction equations developed for maize plants. A modified partial least squares approach implemented in OPUS 6.0 Bruker software was used to fit the calibration equations (Shenk and Westerhaus, 1991). The coefficients of determination for cross-validation (R_CV_^2^) and external validation (R_V al_^2^) were 90.5% and 92.7% for LIG, 94.0% and 96.7% for CEL, 93.6% and 94.6% for ADF, 95.3% and 96.5% for NDF, and 90.2% and 91.2% for IVDMD, respectively.

Because CEL and LIG are components of the cell wall, they were expressed as the percentage of NDF in the QTL analysis (CEL/NDF, LIG/NDF). In addition to the traits mentioned above, cell wall digestibility was investigated according to Struik (1983) and Dolstra and Medema (1990). The IVNDFD was estimated with the following formula: IVNDFD = 100 × (IVDMD-(100-NDF)/NDF, assuming that the non-NDF part of the plant was completely digested.

### Phenotypic Data Analyses

All statistical analyses were performed using R software, version 3.3.2^1^. To eliminate the environmental effects resulting from multiple environments, we fitted a mixed linear model to calculate the best linear unbiased prediction (BLUP) value for each line: y_i_ = μ + g_i_ + e_i_ + 𝜀_i_. In this equation, *y_i_* represents the phenotype of the “*i*”th line, μ is the grand mean value of the target trait in all environments, *g_i_* represents the genetic effect, *e_i_* is the environmental effect (replications in each environment were also treated as environmental effects in the BLUP mixed model), and 𝜀_i_ is the random error. The grand mean was fitted as a fixed effect, and genotype and environment were considered as random effects. The estimated BLUP, which was obtained using the linear mixed effect function “lmer” in the “lme4” package of R, was denoted as the sum of the grand mean and genetic effects of each line. The BLUP values of each line were used as the phenotypic values for QTL mapping.

The aov function in R was used to dissect the phenotypic variance in different environments. The model used for the analysis of variance was y_ilk_ = μ + e_l_ + r_k(l)_ + f_i_ + (fe)_il_ + 𝜀_ilk_, where *e_l_* is the environmental effect of the “*l*”th environment, r_k(l)_ is the effect of replications within environments, *f_i_* represents the genetic effect of the “*i*”th line, (*fe*)*_il_* is the interaction effect between genetic and environment effects, and 𝜀_ilk_ is the residual error. All of the effects were considered to be random. Broad sense heritability was calculated as $h^{2}=\sigma_{g}^{2}/(\sigma_{g}^{2}+\sigma_{ge}^{2}+\sigma_{\varepsilon }^{2}/re)$, where $\sigma_{g}^{2}$ represents the genetic variance, $\sigma_{\mathrm{ge}}^{2}$ is the variance of interaction between the genotype and the environments, $\sigma_{\varepsilon }^{2}$ is the residual error variance item, and *e* and *r* are the number of environments and replications in each environment, respectively. The 95% confidence intervals of the *h*^2^ were calculated following the method of Knapp et al. (1985).

### Genotyping and Genetic Map Construction

Leaf tissues were collected from all 220 RILs and their parents and freeze-dried at -60°C. Genomic DNA was extracted using the modified CTAB method (Murray and Thompson, 1980) and used for genotyping with the MaizeSNP3K DNA-Chip, a subset of the Illumina MaizeSNP50 BeadChip (Ganal et al., 2011) that contains 3,072 SNPs. Single nucleotide polymorphism genotyping was performed on the Illumina GoldenGate SNP genotyping platform (Fan et al., 2006) at the National Maize Improvement Center of China, China Agricultural University. The quality of each SNP was manually controlled as described by Yan et al. (2010), and SNPs with poor quality were excluded from further analysis.

PLINK (Purcell et al., 2007) was used to estimate the minor allele frequency (MAF), missing rate, and heterozygosity for each SNP as well as the missing rate and heterozygosity for each line. After quality control, the SNPs with a missing rate ≤20%, heterozygosity ≤10%, and MAFs ≥0.05 were used to construct the genetic linkage map with QTL ICI-Mapping V3.2 (Meng et al., 2015). Linkage groups were established with the QTL IciMapping software V3.2^2^. The polymorphism markers between two parents were grouped with a minimum logarithm of the odds (LODs) of 8.0. Recombination frequencies were converted into centimorgans using the Kosambi mapping function (Kosambi, 2011). Ordering and rippling procedures were performed with the “nnTwoOpt” algorithm and “the sum of adjacent recombination frequencies (SARFs).”

### QTL Mapping

Best linear unbiased prediction values across environments were used in QTL mapping of the cell wall components and digestibility traits. A whole genome scan was performed using composite interval mapping (Zeng, 1994) implemented in Windows QTL Cartographer 2.5 (Basten et al., 2005). The scanning interval between markers was set at 0.5 cM, and the window size was set at 10 cM. Model 6 of the Zmapqtl module was selected for detecting QTLs and estimating their effects. A forward–backward stepwise regression with five controlling markers was used to control the background signals from flanking markers. The threshold LOD values used to declare the putative QTLs were estimated by permutation tests with a minimum of 1,000 replicates at a significance level of *p* < 0.05 (Churchill and Doerge, 1994). The confidence intervals for the locations of the QTLs were determined via one-LOD support intervals to each side of the position of the maximum LOD (Lander and Botstein, 1989). To estimate the percentage of the phenotypic variance explained by all QTLs, multiple-interval mapping was performed using the Bayesian Information Criteria (BIC-M0) in Windows QTL Cartographer 2.5 (Kao et al., 2004).

## Results

### Phenotypic Variability and Heritability

**FIGURE 1:**
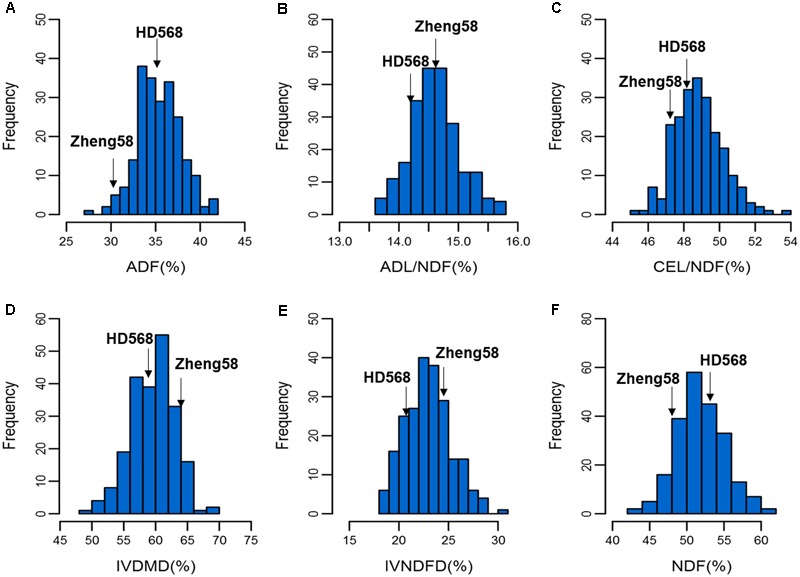
Distribution of cell wall components and digestibility in the RIL population. The frequency distributions of ADF, ADL/NDF, CEL/NDF, IVDMD, IVNDFD, and NDF in the RIL population are shown in **(A–F)**, respectively.

Among the six investigated traits, significant differences were observed between the two parental lines only in IVNDFD and IVDMD. According to the BLUP values of the parental lines, Zheng58 showed higher digestibility (IVNDFD and IVDMD) than HD568 (**Table 1**). The means of the RIL population were close to the mid-parent values for all measured traits. A normal distribution was observed for each trait (**Figure 1**). The ANOVA results showed that both genotype and environmental effects significantly affected the cell wall components and digestibility (*p* < 0.01). Based on the mean square (MS) values, genotype × environment interaction effects were lower than genotype effects for all traits (**Table 1**). Broad sense heritability (*H*^2^) estimates were moderate for all investigated traits and ranged from 0.43 to 0.70.

**Table 1:** Phenotypic variation of cell wall components and digestibility traits in the RIL population.

Traits	Zheng58^a^	HD568^a^	Mean	Max	Min	Geno^b^	Geno × Env^c^	*h*^2d^	CI^e^
ADF	31.89	35.90	35.36 ± 2.44	41.88	27.84	54.82^∗∗^	20.00^∗∗^	0.64	0.52–0.72
ADL/NDF	14.65	14.45	14.61 ± 0.41	15.77	13.71	2.66^∗∗^	1.37^∗∗^	0.49	0.33–0.61
CEL/NDF	47.39	48.71	48.80 ± 1.35	53.69	45.08	23.92^∗∗^	11.73^∗∗^	0.51	0.36–0.62
IVDMD	63.19	58.17	59.53 ± 3.42	69.95	49.38	106.81^∗∗^	23.74^∗∗^	0.63	0.52–0.72
IVNDFD	24.26	21.24	22.98 ± 2.31	30.56	18.18	101.13^∗∗^	57.73^∗∗^	0.43	0.25–0.56
NDF	48.04	52.17	51.80 ± 3.21	60.57	43.32	77.93^∗∗^	39.10^∗∗^	0.70	0.60–0.77

*^a^BLUP value for each parent. ^b^Mean square for genotype. ^c^Mean square for genotype–environment interaction. ^d^Broad-sense heritability. ^e^95% confidence interval of broad-sense heritability. ^∗∗^Significant at *p* < 0.01.*

### Genetic Linkage Map and QTL Analysis

After quality control, 1,358 SNP markers were used to construct the genetic map. The total length of the linkage map for the RIL population was 1,985.6 cM. The average interval of the whole genome scale was 1.5 cM and ranged from 1.0 to 2.2 cM across 10 chromosomes (**Supplementary Data Sheet 1**).

**FIGURE 2:**
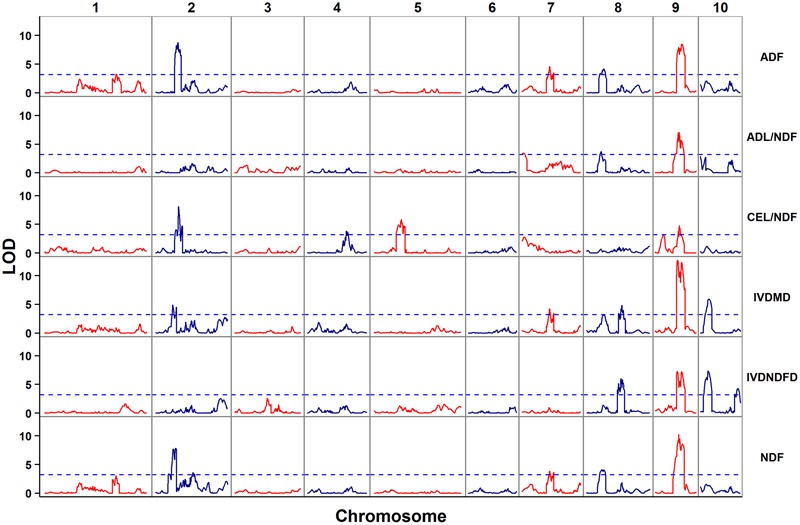
Distribution of putative QTL for cell wall components and digestibility in the RIL population.

The empirical threshold LOD values for the genome-wide significance (*p* < 0.05) were determined to be 3.2 for digestibility traits (IVNDFD and IVDMD) and 3.3 for other four traits after 1,000 permutations. In total, 47 QTL controlling cell wall components and digestibility traits were detected; these QTL corresponded to 10 genomic regions due to the co-localization of different traits (**Table 2** and **Figure 2**). These loci were distributed across eight chromosomes except 3 and 6. Each QTL explained between 4.2 (ADF) and 18.9% (IVDMD) of the phenotypic variation. All additive QTLs mapped for each trait accounted for a moderate proportion of the total phenotypic variation, which ranged from 25.5 (ADL/NDF) to 47.4% (IVDMD) (**Table 2**).

**Table 2:** Summary of QTL for cell wall components and digestibility traits in the RIL population.

Trait	QTL^a^	Chr	Bin	Marker interval	Genetic interval (cM)	Position (cM)^b^	LOD	Additive effect^c^	*R*^2^ (%)^d^
ADF	*adf1*	1	1.07	SYN31271-PZE-101201822	220.61–224.74	222.5	3.29	-0.50	4.2
	*adf2*	2	2.07	PZE-102141193-PZE-102145703	62.73–71.4	69.2	8.76	0.86	11.8
	*adf7*	7	7.03	PZE-107089819-PUT-163a-76010550-3720	84.33–86.77	86.5	4.56	-0.60	5.9
	*adf8*	8	8.01	SYN10430-PZE-108010327	37.69–50.32	53.3	4.17	-0.62	6.4
	*adf9-1*	9	9.02	SYN29878-PZE-109045575	66.73–75.35	73.3	7.94	-0.81	11.1
	*adf9-2*	9	9.03	PZE-109026030-PZE-109076761	80.53–90.3	83.8	8.5	-0.85	12.3
Total^e^									43.96
ADL/NDF	*adl7*	7	7.05	PZE-107132828-SYN34644	0–15.79	5.0	3.46	-0.11	6.4
	*adl8*	8	8.01	SYN10430-PZE-108009277	37.69–46.26	44.3	3.71	0.10	5.7
	*adl9-1*	9	9.02	SYN29878-PZE-109049079	66.73–73.55	72.1	7.02	0.14	11.1
	*adl9-2*	9	9.03	PZE-109036560-PZE-109056180	73.79–76.08	75.1	7.04	0.14	11.1
	*adl9-3*	9	9.03	PZE-109023988-PZE-109063957	79.79–81.79	80.5	5.69	0.13	9.1
Total^e^									25.5
CEL/NDF	*cel2-1*	2	2.07	SYN5428-PUT-163a-71763840-3475	58.99–67.26	63.7	4.12	0.36	6.4
	*cel2-2*	2	2.07	PZE-102139681-PZE-102142740	69.2–73.4	71.4	8.12	0.49	11.6
	*cel2-3*	2	2.08	PZE-102126078-PZE-102132750	79.81–83.88	82.6	4.72	0.38	7.4
	*cel4-1*	4	4.04	PZE-104024382-SYN8382	119.9–123.56	120.9	3.83	0.32	5.3
	*cel4-2*	4	4.04	PZE-104021665-PZE-104024382	123.56–126.48	126	3.61	0.32	5
	*cel5-1*	5	5.03	SYN30418-PZE-105047885	80.59–87.88	83.6	5.84	-0.40	8.2
	*cel5-2*	5	5.03	SYN27691-PZE-105051986	89.33–95.62	90.6	4.71	-0.35	6.7
	*cel9-1*	9	9.02	SYN29878-PZE-109049079	66.73–73.55	72.1	3.8	0.34	5.3
	*cel9-2*	9	9.03	PZE-109036560-PZE-109056255	73.79–78.07	75.1	4.75	0.37	6.5
	*cel9-3*	9	9.03	PZE-109026030-PZE-109063957	79.79–80.53	80.5	3.35	0.31	4.7
Total^e^									34.9
IVDMD	*ivdmd2-1*	2	2.07	PZE-102147840-PZE-102154251	48.12–54.71	53.3	4.88	-0.86	6.1
	*ivdmd2-2*	2	2.07	PZE-102145606-PZE-102145703	62.73–64.89	64.7	4.49	-0.82	5.7
	*ivdmd7-1*	7	7.03	PZE-107089819-PUT-163a-76010550-3720	84.33–86.77	85.6	4.24	0.82	5.7
	*ivdmd7-2*	7	7.03	PZE-107077981-PZA01714.1	95.82–98.86	98.8	3.47	0.73	4.5
	*ivdmd8-1*	8	8.04	PZE-108067466-SYN34824	99.62–101.27	100.6	3.3	0.72	4.3
	*ivdmd8-2*	8	8.05	PZE-108074258-SYN18235	105.81–111.12	109	4.87	0.85	6.1
	*ivdmd9-1*	9	9.02	SYN29878-PZE-109027216	66.73–72.07	69.7	12.69	1.50	18.9
	*ivdmd9-2*	9	9.03	PZE-109023988-PZE-109026030	80.53–81.79	81.8	12.33	1.41	16.9
	*ivdmd10*	10	10.07	PZE-110105621-SYN22564	21.6–23.28	26.3	5.9	-0.99	8.2
Total^e^									47.4
IVNDFD	*ivndfd8-1*	8	8.04	PZE-108066888-PZE-108067466	97.76–99.62	98.7	5.17	0.63	7
	*ivndfd8-2*	8	8.05	PZE-108075114-PZE-108079027	105.58–109.43	106.3	6.03	0.68	8.1
	*ivndfd8-3*	8	8.05	PZE-108079422-PZE-108083054	109.67–112.81	110.9	5.83	0.67	8
	*ivndfd9-1*	9	9.02	PZE-109016787-SYN29878	55.67–66.73	68.7	7.28	0.80	11.2
	*ivndfd9-2*	9	9.03	PZE-109036560-PZE-109037929	73.79–79.31	77.1	5.53	0.66	7.9
	*ivndfd9-3*	9	9.03	PZE-109023988-PZE-109026030	80.53–81.79	81.8	7.24	0.74	9.9
	*ivndfd10-1*	10	10.07	PZE-110105621-SYN22564	21.6–23.28	24.3	7.29	-0.76	10.4
	*ivndfd10-2*	10	10.02	PZE-110008811-PZE-110010390	110.12–116.21	117.2	4.31	-0.60	6.3
Total^e^									39.1
NDF	*ndf2-1*	2	2.09	SYN7501-PZE-102168198	34.18–39.17	41.2	3.36	0.73	5.2
	*ndf2-2*	2	2.08	PZE-102147840-PZE-102154251	48.12–54.71	54.7	7.69	1.05	10.2
	*ndf2-3*	2	2.08	PZE-102145606-PUT-163a-71763840-3475	58.99–64.89	62	7.84	1.11	11.3
	*ndf2-4*	2	2.04	PZE-102063830-PZE0006607533	114.75–116.93	116.5	3.62	0.77	4.3
	*ndf7-1*	7	7.03	PZE-107089819-SYN32262	84.56–86.77	86.5	3.82	-0.72	4.9
	*ndf7-2*	7	7.03	PZE-107077981-PZA01714.1	95.82–98.86	98.8	3.64	-0.70	4.7
	*ndf8*	8	8.01	SYN10430-PZE-108010327	37.69–50.32	47.3	4.13	-0.76	5.5
	*ndf9-1*	9	9.03	PZE-109027216-PZE-109049079	72.07–73.55	73.3	10.27	-1.22	14.2
	*ndf9-2*	9	9.03	PZE-109026030-PZE-109076761	80.53–90.3	84.8	8.57	-1.15	12.7
Total^e^									41.7

*^a^Each QTL was named with its associated trait name and chromosomal location. Different numbers following the dash indicate different QTL on the same chromosome. ^b^Peak position with the greatest LOD. ^c^Additive effect of the identified QTL: a positive value indicates that the Z58 allele increased trait expression and a negative value indicates that the HD568 allele increased expression. ^d^Phenotypic variation explained by the additive effects of the mapped QTL. ^e^Total percentage of phenotypic variation explained by all additive effects of the mapped QTL for each trait.*

For ADF content, six QTL were detected on chromosomes 1, 2, 7, 8, and 9. Among these QTL, *adf9-2*, with the largest effect (12.3% of the phenotypic variation) was located on chromosome 9. The HD568 allele at this locus contributed an additive effect of 0.85% for ADF concentration. Another important locus for ADF content, *adf9-1*, which explained 11.1% of the phenotypic variation with an additive of 0.81%, was also located on chromosome 9. Moreover, another major effect QTL (*adf2*) that explained more than 10% of the phenotypic variation was found on chromosome 2. The allele from Zheng58 at this locus was associated with an increase in ADF content.

Five QTL were significantly associated with ADL/NDF. These QTL were distributed on chromosomes 7, 8, and 9 and explained 25.5% of the total phenotypic variation. A QTL cluster detected on chromosome 9 consisted of three adjacent QTL that explained 11.1, 11.1, and 9.1% of the phenotypic variation, respectively. With the exception of *adl7*, the alleles of these QTLs that increased ADL/NDF came from Zheng58.

A total of 10 QTL controlling CEL/NDF that explained 34.9% of the total phenotypic variation were distributed on chromosomes 2, 4, 5, and 9. These QTL were located in four genomic regions. The strongest QTL, *cel2-2*, explained 11.6% of the phenotypic variation, and the Zheng58 allele had an additive effect of 0.49% for increasing CEL/NDF. The phenotypic variance explained by the other nine QTL ranged from 4.7 to 7.4%. With the exception of two QTL on chromosome 5 (*cel5-1* and *cel5-2*), the alleles of these nine QTL with increasing effects came from Zheng58.

For IVDMD, nine additive effect QTL that explained 47.4% of the total phenotypic variation were located on chromosomes 2, 7, 8, 9, and 10. Among all the identified QTLs for IVDMD, two QTL on chromosome 9 accounted for >15% of the phenotypic variation (**Table 2**). The other QTL explained only 4.3–8.2% of the phenotypic variation. HD568 alleles at three loci, *ivdmd2-1, ivdmd2-2*, and *ivdmd10*, contributed to increased IVDMD.

Eight QTL were associated with IVNDFD and accounted for 39.1% of the total phenotypic variation. These QTL were located on chromosomes 8, 9, and 10 and explained 6.3–11.2% of the variation. With the exception of *ivndfd10-1* and *ivndfd10-*2, the alleles of these eight QTL with increasing effects came from Zheng58.

Four out of nine QTL for NDF accounted for >10% of the phenotypic variation. The strongest QTL, *ndf9-1*, which was flanked by the markers PZE-109027216 and PZE-109049079, explained 14.2% of the phenotypic variation. Four QTL were located on chromosome 2, and Zheng58 alleles at these loci had additive effects that increased NDF. The alleles of the other five QTL with increasing effects came from HD568. Collectively, the nine QTL for NDF explained 41.7% of the total phenotypic variation.

In addition to the individual additive QTL, five pairs of epistatic QTL involving nine loci were identified for ADF, CEL/NDF, IVNDFD, and NDF (**Table 3**). The proportion of total phenotypic variance explained by all epistatic QTL ranged from 2.2 to 4.7%. With the exception of *ivndfd10-1* and *ivndfd10-2*, the combination of alleles at all interacting loci with increasing contributions were inherited from different parents.

**Table 3:** Epistatic QTL for cell wall components and digestibility traits in the RIL population.

Trait	QTL_i	Chr_i	Marker interval_i	p_i^a^	QTL_j	Chr_j	Marker interval_j	p_j^a^	AA^b^	*R*^2^ (%)^c^
ADF	*adf2*	2	PZE-102141193-PZE-102145703	69.2	*adf8*	8	SYN10430-PZE-108010327	53.3	-0.33	2.6
CEL/NDF	*cel2-1*	2	SYN5428-PUT-163a-71763840-3475	63.7	*cel2-2*	2	PZE-102139681-PZE-102142740	71.4	-0.35	2.2
IVNDFD	*ivndfd8-2*	8	PZE-108075114-PZE-108079027	106.3	*ivndfd10-1*	10	PZE-110105621-SYN22564	24.3	-0.46	4.7
IVNDFD	*ivndfd10-1*	10	PZE-110105621-SYN22564	24.3	*ivndfd10-2*	10	PZE-110008811-PZE-110010390	117.2	0.47	3.9
NDF	*ndf2-3*	2	PZE-102145606-PUT-163a-71763840-3475	62	*ndf8*	8	SYN10430-PZE-108010327	47.3	-0.67	4.6

*^a^Genetic position of each QTL was estimated by multiple interval mapping. ^b^Positive (+) means that parental digenic genotypes increase trait expression and negative (-) means that the recombinant alleles from both parents increase trait expression. ^c^Percentage of phenotypic variance explained by individual epistatic effects of the mapped QTL.*

### Co-localization of Individual QTL for Each Trait

A comparison of the QTL for different traits revealed a conspicuous QTL hotspot on chromosome 9. This QTL cluster was flanked by SNP markers PZE-109016787 and PZE-109076761 and had a genetic interval from 55.7 to 90.3 cM. Two–three QTL for each trait were found in this genomic region. The QTL located within this genomic region contributed a large proportion (ranging from 4.7 to 18.9%) of the phenotypic variation for each trait. The QTL for ADF, ADL/NDF, and NDF on chromosome 8 (*adf8, adl8*, and *ndf8*) shared a common left flanking marker, SYN10430. Moreover, the LOD score plot for chromosome 8 (**Figure 2**) showed another potential QTL peak for IVDMD within this overlapped QTL region, though without significance (LOD ≥ 3.3). The additive effect QTL on chromosome 2 were found sharing overlapping confidence interval and explained 11.8, 5.7, and 11.3% of the phenotypic variation of ADF, IVDMD, and NDF, respectively (**Figure 2**). The overlapped region of these QTL spanned a 2.2 cM genetic distance and ranged from 62.7 to 64.9 cM. In addition to chromosome 2, overlapping QTL for ADF, IVDMD, and NDF were also detected on chromosome 7 and ranged from 84.6 to 86.8 cM.

## Discussion

### Phenotypic Variation and Heritability

In the present study, a panel consisting of 220 RILs was used to determine the genetic architecture of cell wall components and digestibility. Based on the BLUP values of two parents across different environments, HD568 showed relatively higher ADF and NDF contents and lower forage and cell wall digestibility compared with Zheng58 (**Table 1**). Transgressive segregation for each trait was observed in the RILs (**Figure 1**), which could be attributed to the pyramiding of advantageous alleles at different loci. The ANOVA results showed that environmental and genetic effects played important roles in affecting phenotypic variance. Previous studies have shown significant variations in the broad sense heritability of each cell wall component and digestibility trait, which ranged from 0.49 to 0.96 (Lübberstedt et al., 1997a,b; Bohn et al., 2000; Barriere et al., 2001; Méchin et al., 2001; Papst et al., 2001; Roussel et al., 2002; Cardinal et al., 2003; Fontaine et al., 2003; Krakowsky et al., 2003, 2005, 2006; Barrière et al., 2007; Riboulet et al., 2008). As in previous studies, the broad sense heritability estimates of the present study were moderate for the QTL analysis, ranging from 0.43 (IVNDFD) to 0.70 (NDF). The significant variation of heritability between different studies demonstrates the complexity of the genetic architecture of cell wall components and digestibility. Further characterization of these traits in additional bi-parental segregating populations is needed to reconcile these differences in heritability values.

### Genetic Architecture of Maize Cell Wall Components and Digestibility at the Anthesis Stage

Thus far, numerous QTL related to different cell wall components and digestibility traits in maize have been investigated in previous studies (Lübberstedt et al., 1997a,b; Bohn et al., 2000; Barriere et al., 2001; Méchin et al., 2001; Papst et al., 2001; Roussel et al., 2002; Cardinal et al., 2003; Fontaine et al., 2003; Krakowsky et al., 2003, 2005, 2006; Barrière et al., 2007; Riboulet et al., 2008). These QTL can affect the phenotypic variations of silage quality and cell wall-related traits. Among these QTL mapping studies, three of them were performed at anthesis stage (Krakowsky et al., 2003, 2005, 2006). In the present study, 47 QTL were detected in the RIL population, with 4–10 QTL for each trait explaining 4.2–18.9% of the phenotypic variation in the Zheng58 × HD568 RIL population (**Figure 2** and **Table 2**). Among these QTL, 29.8% could explain more than 10% of the phenotypic variation. All these findings together with previous QTL mapping studies about cell wall components and digestibility traits revealed that a few major effect QTLs and some minor effect QTLs provide most of the genetic bases of cell wall components and digestibility in maize.

The low resolution caused by the low density of RFLP or SSR markers in previous studies makes co-localization likely for most QTLs detected in the current study.

In addition to additive effect QTL, five pairs of epistatic QTL were also detected for ADF, CEL/NDF, IVNDFD, and NDF in our study. The majority of these pairs of epistatic QTL explained less than 10% of the phenotypic variations of each trait. These results suggest that epistasis also contributes to the complex nature of the cell wall components and digestibility of maize stalks, though the effects of epistasis are relatively weak.

Compared with previous QTL mapping studies of cell wall components at the anthesis stage, overlapping QTL were observed on bin 1.07 for ADF, bin 2.04 and 2.08 for NDF, and bin 9.03 for LIG in the present study (Krakowsky et al., 2003, 2005, 2006). Is the same gene playing roles in these overlapped QTL should be evaluated with fine mapping and more genetic approaches. A few co-localizations were also found between the current study and previous QTL mapping studies performed at silage stage, which included bin 9.02 for LIG content, IVDMD, and IVNDFD, bin 8.05 for IVDMD, and so on. The low resolution caused by the low density of RFLP or SSR markers in previous studies makes co-localization likely for some QTLs detected in the current study. These results revealed that a few genes are involved in cell wall biosynthesis consistently between anthesis stage and silage stage, and most genes that function in cell wall component accumulation have temporal specificity.

### Hotspots of QTL for Cell Wall Components and Digestibility

Due to the significant correlation between cell wall traits and digestibility, it is easy to predict their genetic correlations and their co-localization in the genome. In a previous meta-analysis study, several regions were highlighted as hotspots of QTL for cell wall components and digestibility traits; these hotspots included bins 1.08, 1.11, 2.08, 3.07, 4.04, 5.04, 9.01, and 9.07 (Truntzler et al., 2010). In the current study, a QTL hotspot related to all six investigated traits was found on chromosome 9, which covers a large part of the maize genome of bin 9.02–9.03. Moreover, three regions (localized in bins 2.07, 7.03, and 8.01–8.02) were identified as overlapping QTL for several of the traits studied herein. These regions will be further investigated by association mapping or fine mapping to identify variants correlated with cell wall components and digestibility traits. This approach should be helpful in determining whether these overlapped regions were caused by pleiotropy, improve our understanding of cell wall biosynthesis, and provide molecular markers for improving forage quality.

## Availability of Data and Materials

The datasets supporting the conclusions of this article are included in the article and in its additional files.

## Author Contributions

KL and HW carried out the experiments, analyzed the data, and wrote the manuscript; XH and YW assisted in data collection and analysis; FM and QW designed and performed the field experiments; and CH and ZL designed the study and helped to write the manuscript. All authors have read and approved the final version of the manuscript.

## Conflict of Interest Statement

The authors declare that the research was conducted in the absence of any commercial or financial relationships that could be construed as a potential conflict of interest.

## Footnotes

1 http://www.R-project.org

2 http://www.isbreeding.net/software/default.aspx?type=list&class=20

## Supplementary Material

DATA SHEET 1 Summary of genetic map of the RIL population.
